# Emotional sounds modulate early neural processing of emotional pictures

**DOI:** 10.3389/fpsyg.2013.00741

**Published:** 2013-10-18

**Authors:** Antje B. M. Gerdes, Matthias J. Wieser, Florian Bublatzky, Anita Kusay, Michael M. Plichta, Georg W. Alpers

**Affiliations:** ^1^Department of Psychology, School of Social Sciences, University of MannheimMannheim, Germany; ^2^Department of Psychology, University of WürzburgWürzburg, Germany; ^3^Department of Psychiatry and Psychotherapy, Central Institute of Mental Health, Medical Faculty Mannheim/Heidelberg UniversityMannheim, Germany; ^4^Otto-Selz Institute, University of MannheimMannheim, Germany

**Keywords:** emotional pictures, emotional sounds, audiovisual stimuli, ERPs, P100, P200, LPP

## Abstract

In our natural environment, emotional information is conveyed by converging visual and auditory information; multimodal integration is of utmost importance. In the laboratory, however, emotion researchers have mostly focused on the examination of unimodal stimuli. Few existing studies on multimodal emotion processing have focused on human communication such as the integration of facial and vocal expressions. Extending the concept of multimodality, the current study examines how the neural processing of emotional pictures is influenced by simultaneously presented sounds. Twenty pleasant, unpleasant, and neutral pictures of complex scenes were presented to 22 healthy participants. On the critical trials these pictures were paired with pleasant, unpleasant, and neutral sounds. Sound presentation started 500 ms before picture onset and each stimulus presentation lasted for 2 s. EEG was recorded from 64 channels and ERP analyses focused on the picture onset. In addition, valence and arousal ratings were obtained. Previous findings for the neural processing of emotional pictures were replicated. Specifically, unpleasant compared to neutral pictures were associated with an increased parietal P200 and a more pronounced centroparietal late positive potential (LPP), independent of the accompanying sound valence. For audiovisual stimulation, increased parietal P100 and P200 were found in response to all pictures which were accompanied by unpleasant or pleasant sounds compared to pictures with neutral sounds. Most importantly, incongruent audiovisual pairs of unpleasant pictures and pleasant sounds enhanced parietal P100 and P200 compared to pairings with congruent sounds. Taken together, the present findings indicate that emotional sounds modulate early stages of visual processing and, therefore, provide an avenue by which multimodal experience may enhance perception.

## Introduction

In everyday life people are confronted with an abundance of different emotional stimuli from the environment. Typically, these cues are transmitted through multiple sensory channels and especially audiovisual stimuli (e.g., information from face and voice in the social interaction context) are highly prevalent. Only a fraction of this endless stream of information however is consciously recognized, is attended to and more elaborately processed (Schupp et al., 2006). To cope with limited processing capacities, emotionally relevant cues have been suggested to benefit from prioritized information processing (Vuilleumier, 2005). Despite the high relevance of multimodal emotional processing, emotion research has mainly focused on investigating unimodal stimuli (Campanella et al., 2010). Furthermore, existing studies on multimodal stimuli predominantly investigated how emotional faces and emotional voices are integrated (for a recent review see Klasen et al., 2012). As expected, most of the studies generally indicate that behavioral outcome is based on interactive integration of multimodal emotional information (de Gelder and Bertelson, 2003; Mothes-Lasch et al., 2012). For example, emotion recognition is improved in response to redundant multimodal compared to unimodal stimuli (Vroomen et al., 2001; Kreifelts et al., 2007; Paulmann and Pell, 2011). Furthermore, the identification and evaluation of an emotional facial expression is biased toward the valence of simultaneously presented affective prosodic stimuli and vice versa (de Gelder and Vroomen, 2000; de Gelder and Bertelson, 2003; Focker et al., 2011; Rigoulot and Pell, 2012). Such interactions between emotional face and voice processing even occur when subjects were asked to ignore concurrent sensory information (Collignon et al., 2008) and were shown to be independent of attentional resources (Vroomen et al., 2001; Focker et al., 2011). In addition, the processing of emotional cues can even alter responses to non-related events coming from a different sensory modality which may indicate that an emotional context can modulate the excitability of sensory regions (Dominguez-Borras et al., 2009).

Regarding cortical stimulus processing, event-related potentials (ERP) to picture cues are well-suited to investigate the time course of attentional and emotional processes (Schupp et al., 2006). Already early in the visual processing stream, differences have been shown for emotional as compared to neutral pictures for the P100, P200, and the early posterior negativity (EPN). These early components may relate to facilitated sensory processing fostering detection and categorization processes. Later processing stages have been associated with detailed evaluation of emotional visual cues (e.g., the late positive potential, LPP). The P100 component indexes early sensory processing within the visual cortex, which is modulated by spatial attention and may reflect a sensory gain control mechanisms to attended stimuli (Luck et al., 2000). Studies on emotion processing have reported enhanced P100 amplitudes for unpleasant pictures and threatening conditions—but also for pleasant stimuli which has been interpreted as an early attentional orientation toward emotional cues (see e.g., Pourtois et al., 2004; Brosch et al., 2008; Bublatzky and Schupp, 2012). Further, as an indicator of early selective stimulus encoding the EPN has been related to stimulus arousal for both pleasant and unpleasant picture materials (Schupp et al., 2004). In addition, the P200 has been considered as an index of affective picture processing (Carretie et al., 2001a, 2004). Enhanced P200 amplitudes in response to unpleasant and pleasant cues suggest that emotional cues mobilize automatic attention resources (Carretie et al., 2004; Delplanque et al., 2004; Olofsson and Polich, 2007). In addition to affective scenes, enhanced P200 amplitudes were also reported for emotional words (e.g., Kanske and Kotz, 2007) and facial expressions (Eimer et al., 2003). Subsequent in the visual processing stream, the LPP over centro-parietal sensors (developing around 300 ms after stimulus onset) is sensitive for emotional intensity (Cuthbert et al., 2000; Schupp et al., 2000; Bradley et al., 2001). Further, the LPP has been associated to working memory and competing tasks indicating the operation of capacity-limited processing (for a review see Schupp et al., 2006). Taken together, affect-modulation of visual ERPs can be identified at both early and later processing stages.

Research on multimodal integration of emotional faces and voices has also reported an early modulation of ERP components (i.e., around 100 ms poststimulus). These effects have been interpreted as evidence for an early influence of one modality on the other (de Gelder et al., 1999; Pourtois et al., 2000; Liu et al., 2012). Comparing unimodal and multimodal presentations of human communication, Stekelenburg and Vroomen (2007) observed an effect of multimodality on the N100 and the P200 component time-locked to the sound onset. They report a decrease in amplitude and latency for the presentation of congruent auditory and visual human stimuli compared to unimodally presented sounds. Likewise, Paulmann et al. (2009) suggested that an advantage of congruent multimodal human communication cues compared to unimodal auditory perception is reflected by a systematic decrease of P200 and N300 components. In a recent study, videos of facial expressions and body language with and without emotionally congruent human sounds were investigated (Jessen and Kotz, 2011). Focusing on auditory processing, the N100 amplitude was strongly reduced in the audiovisual compared to the auditory condition, indicating a significant impact of visual information on early auditory processing. Further, simultaneously presented congruent emotional face-voice combinations elicited enhanced P200 and P300 amplitudes for emotional relative to neutral audiovisual stimuli, irrespective of valence (Liu et al., 2012). Taken together, these studies support the notion that audiovisual compared to unimodal stimulation is characterized by reduced and speeded processing effort.

Regarding the match or mismatch of emotional information from different sensory channels, differences in ERPs to congruent and incongruent information have been reported. De Gelder et al. (1999) presented angry voices with congruent (angry) or incongruent (sad) faces and observed a mismatch negativity effect (MMN) around 180 ms after stimulus onset for incongruent compared to congruent combinations. Likewise, Pourtois et al. (2000) investigated multimodal integration with congruent and incongruent pairings of emotional facial expression and emotional prosody. They reported delayed auditory processing for the incongruent condition as indexed by a delayed posterior P2b component in response to incongruent compared to congruent face-voice-trials (Pourtois et al., 2002).

Beyond face-voice integration, there are only very few studies, which investigated interactions of emotional picture and sound stimuli. On the one hand, there are some studies which included bodily gestures to investigate multimodal interactions—see above (Stekelenburg and Vroomen, 2007; Jessen and Kotz, 2011; Jessen et al., 2012), on the other side, there are studies investigating interactions between musical and visual stimuli (Baumgartner et al., 2006a,b; Logeswaran and Bhattacharya, 2009; Marin et al., 2012). For instance, music can enhance the emotional experience of emotional pictures (Baumgartner et al., 2006a). Combined (congruent) presentation of pictures and music enhanced peripherphysiological responses and evoked stronger cortical activation (alpha density) in comparison to unimodal presentations. Similarly, presenting congruent or incongruent pairs of complex affective pictures and affective human sounds led to an increased P200 as well as an enhanced LPP in response to congruent compared to incongruent stimulus pairs (Spreckelmeyer et al., 2006). Thus, multimodal simultaneity is not limited to human communication.

Building upon these findings, the present study examines how picture processing is influenced by simultaneously presented complex emotional sounds (e.g., sounds of a car crash, laughing children). We did not aim at optimizing mutual influences by semantic matches of related audiovisual stimulus pairs (such as the picture and the sound of an accident), instead, we wanted to examine the interaction of valence-specific pairs (such as the sight of a child and the sound of a crash). Overall, based on previous findings we expect that emotional information of one modality modulate the EEG components in response to the other modality. Specifically, we expect that the presentation of emotional sounds modulate early as well as later processing stages of visual processing. It is expected that picture processing is generally affected by a concurrent sound compared to pictures only. Furthermore, emotional sounds should differentially modulate visual processing according to their congruence or incongruence to the emotional content of the pictures.

## Materials and methods

### Participants

Participants were recruited from the University of Mannheim as well as via personal inquiry and advertisements in local newspapers. The group consisted of 22 participants^1^ (11 female) with a mean age of *M* = 21.32, *SD* = 2.85. Participation in the study was voluntary and students received class credits for participation. External participants received a small gift, but no financial reimbursement. The study protocol was approved by the ethics committee of the University of Mannheim.

Exclusion criteria included any severe physical illness as well as current psychiatric or neurological disorder and depression as indicated by a score of 39 or higher on the German version of the Self-Rating Depression Scale [SDS, CIPS (1986)]. Also participants reported normal or corrected-to-normal vision and audition and no use of psychopharmaca. In addition, the following questionnaires were completed: a personal data form, the German version of the SDS (*M* = 31.48, *SD* = 4.05), the German version of the Positive and Negative Affect Schedule (Positive affect: *M* = 30.90, *SD* = 5.66, Negative affect: *M* = 11.14, *SD* = 1.11, Krohne et al., 1996), as well as the German Version of the State-Trait-Anxiety Inventory (Trait version: *M* = 33.95, *SD* = 6.90, State: *M* = 30.62, *SD* = 3.94, Laux et al., 1981)^2^.

### Stimulus materials

The stimulus material consisted of 20 pleasant, 20 unpleasant, and 20 neutral pictures selected from the International Affective Picture System (Lang et al., 2008) as well as the same amount of pleasant, unpleasant and neutral sounds selected from the International Affective Digitalized Sounds database (Bradley and Lang, 2007)^3^. Stimuli were selected for comparable valence and arousal ratings between pleasant and unpleasant stimuli and between pictures and sounds. Furthermore, different content categories (human, animals, inanimate) were represented in the most balanced way possible between the valence categories as well as between sound and pictures. The original sound stimuli of the IADS were cut to a duration of 2 s and used in this edited version^4^ (see also Noulhiane et al., 2007; Mella et al., 2011).

### Experimental procedure

Upon arrival in the laboratory the location and procedure were introduced and participants read and signed the informed consent form. The electrode cap and electrodes were then attached. Afterwards, participants were seated on a chair approximately 100 cm away from the monitor (resolution: 1280 × 960 pixel) in the separate EEG booth and were asked to fill in the questionnaires. Upon finishing the preparation phase, participants were informed about the procedure and instructed to view the pictures presented on the computer monitor and listen to the sounds presented through headphones (AKG K77). Also they were told to move as little as possible. Practice trials were presented in order to customize participants to the procedure before the main experiment was started. Overall, the experimental part consisted of 60 visual (pictures only) and 180 audiovisual trials^5^. Visual and audiovisual trials were presented in randomized order.

During visual trials, 20 pleasant, 20 neutral, and 20 unpleasant pictures were displayed for 2 s each. After 50% of the trials 9-point-scales of the Self-Assessment-Manikin (Bradley and Lang, 1994) were presented for ratings of valence and arousal. To shorten the experimental procedure, the participants rated only 50% of all stimulus presentations. The selection of the stimuli was counterbalanced across participants so that all stimulus presentations were rated by 50% of the participants. In cases of no rating, an interval of 2000 ms followed.

For the audiovisual condition, sounds were presented for 2 s with pictures being presented 500 ms after sound onset with a total duration of also 2 s resulting in an overall trial length of 2.5 s. Again stimuli had to be rated in 50% of the trials and the task was to rate valence and arousal elicited by the combination of both, picture and sound. The sound and picture onset were asynchronous as the grasp of the emotional meaning of a sound is not as precise and clearly defined with the onset as compared to a picture. To ensure that the emotional meaning of the sound was present when the picture was presented, we decided to present the picture after a delay of 500 ms.

Overall, the audiovisual condition consisted of 180 trials. Every picture condition (pleasant, neutral, and unpleasant) was paired with every sound condition (pleasant, neutral and unpleasant). This results in nine different conditions with 20 trials with pleasant pictures and pleasant sounds, 20 trials with unpleasant pictures and unpleasant sounds (congruent), 20 trials with pleasant pictures paired with unpleasant sounds and 20 trials with unpleasant pictures with pleasant sounds (incongruent). Additionally, pleasant, unpleasant and neutral pictures were paired each with neutral sounds (60 trials) as well as pleasant and unpleasant sounds with neutral pictures (40 trials).

Ratings were completed using the corresponding keyboard button. Overall, the experimental session lasted about 45 min.

### Data acquisition and preprocessing

Electrophysiological data were collected with a 64-channel recording system (actiCAP, Brain Products GmbH, Munich) with a sampling rate of 1 kHz. Electrodes were recorded according to the international 10–20-system. FCz served as the reference electrode and AFz as the ground electrode. Scalp impedance was kept below 10 kΩ. Data was recorded with an EEG-amplifier Brain-Amp-MR Amplifier (Brain Products GmbH, Munich, Germany).

EEG-data were offline re-referenced to an average reference and filtered (Notch filter of 50 Hz; IIR filter: high cut-off 30 Hz; low cut-off 0.1 Hz) using Brainvision Analyzer 2 (by Brain Products GmbH). Ocular correction was conducted via a semi-automatic Independent Component Analysis (ICA)-based correction process. For data reduction stimulus-synchronized segments with a total length of 1600 ms lasting from 100 ms before and 1500 ms after picture onset were extracted. These segments were then passed through an automatic Artifact Rejection algorithm also provided by Brainvision Analyzer 2. Artifacts were defined with the following criteria: a voltage step of more than 50.0 μV/ms, a voltage difference of 200 μV within the segments, amplitudes of less than −100 μV or more than 100 μV and a maximum voltage difference of more than 0.50 V within 100-ms intervals.

Afterwards all remaining segments (97.5%) for each condition, sensor and participant were baseline corrected (100 ms before stimulus onset) and averaged to calculate the ERPs from the spontaneous EEG.

### Statistical analysis

#### Self-report data

The affective ratings for valence and arousal were analyzed by separate repeated measure analyses of variance (ANOVAs).

***Visual vs. audiovisual condition***. Within-subject variables were *Modality* (visual vs. audiovisual trials), and *Stimulus Category* (congruent pleasant vs. congruent unpleasant vs. congruent neutral). In terms of comparableness of the visual and audiovisual trials for valence, we only considered congruent audiovisual trials for this analysis.

***Audiovisual condition***. Separate repeated measures ANOVAs for audiovisual trials only were conducted with the within-subject variables *Sound Category* (pleasant vs. unpleasant vs. neutral) and *Picture Category* (pleasant vs. unpleasant vs. neutral).

***Congruency***. To test specific differences between congruent and incongruent trials separately for pleasant and unpleasant pictures, planned *t*-tests were conducted at *p*-value < 0.05.

In order to correct for violations of sphericity the Greenhouse-Geisser corrected *p*-value was used to test for significance. Separate ANOVAs as well as *post-hoc t*-tests (bonferroni-corrected) were used for follow up analyses.

#### Electrophysiological data

As sound stimuli develop their emotional meaning over time and thus, the emotional onset is not clearly defined, ERPs were locked to picture onsets only. Based on visual inspection and previous research, three time windows and sensor areas were identified: for the P100 component, the mean activity in a time window from 90 to 120 ms was averaged over parietal and occipital electrodes (left: P3, O1; right: P4,O2); for the P200, mean activity between 170 and 230 ms was averaged over parietal and central electrodes (left: P3, C3, right P4, C4—see Stekelenburg and Vroomen, 2007) and the LPP was scored at CP1 and CP2 in a time interval ranging from 400 to 600 ms (see Schupp et al., 2000, 2007)^6^.

***Visual vs. audiovisual condition***. To investigate the general influence of the sound presentation on picture processing, mean amplitudes for P100, P200, and LPP were subjected to separate repeated measures analyses of variances (ANOVAs). Within-subject variables were *Modality* (visual vs. audiovisual trials), *Stimulus Category* (congruent pleasant vs. congruent unpleasant vs. congruent neutral), and *Electrode Site*^7^. In terms of comparableness of the visual and audiovisual trials for valence, we only considered congruent audiovisual trials for this analysis.

***Audiovisual condition***. To further examine the influence of the emotional content of the sounds on picture processing and possible interactions of the emotional contents, for the P100, P200, and the LPP separate repeated measures ANOVAs for audiovisual trials only were conducted with the within-subject variables *Sound Category* (pleasant, unpleasant, neutral) and *Picture Category* (pleasant, unpleasant, neutral) and *Electrode Site.*

***Congruency***. To test specific differences between congruent and incongruent trials separately for pleasant and unpleasant pictures, planned *t*-tests were conducted at *p*-value < 0.05.

In order to correct for violations of sphericity the Greenhouse-Geisser corrected *p*-value was used to test for significance (according to Picton et al., 2000). Effects of *Electrode Site* were only considered if they interact with one of the other variables. Separate ANOVAs as well as *post-hoc t*-tests (bonferroni-corrected) were used for follow up analyses.

## Results

### Self-report data

#### Valence

***Visual vs. audiovisual condition***. For the valence ratings a significant main effect of *Stimulus Category, F*_(2, 42)_ = 353.61, *p* < 0.001, η^2^_*p*_ = 0.94, was observed, as well as a significant interaction of *Modality* and *Stimulus Category, F*_(2, 42)_ = 7.01, *p* = 0.003, η^2^_*p*_ = 0.25, but no significant main effect of *Modality*. As expected, unpleasant stimuli were rated as more unpleasant than neutral or pleasant stimuli and pleasant stimuli were rated as most pleasant [unpleasant vs. neutral: *t*_(21)_ = 19.91, *p* < 0.01; pleasant vs. neutral *t*_(21)_ = 13.03, *p* < 0.01; pleasant vs. unpleasant: *t*_(21)_ = 20.41, *p* < 0.01]. Following the interaction, audiovisual pairs with pleasant sounds and pictures were rated as more pleasant than pleasant pictures only, *t*_(21)_ = 3.47, *p* < 0.01, whereas unpleasant sounds with unpleasant pictures were rated as marginally more unpleasant than unpleasant pictures only, *t*_(21)_ = 1.89, *p* < 0.10—see Table 1.

**Table 1:** **Mean and standard deviation for valence and arousal ratings of pleasant, neutral and unpleasant visual and congruent audiovisual presentations**.

**Rating**	**Emotion Category**	**Visual *M* (*SD*)**	**Audiovisual *M* (*SD*)**
Valence	Pleasant	6.87 (0.76)	7.13 (0.65)
	Neutral	5.06 (0.39)	5.00 (0.54)
	Unpleasant	2.36 (0.63)	2.17 (0.59)
Arousal	Pleasant	4.81 (1.12)	5.18 (0.95)
	Neutral	4.30 (1.16)	4.55 (1.14)
	Unpleasant	6.50 (0.92)	6.90 (0.81)

***Audiovisual condition***. Focusing on audiovisual trials only, the ANOVA with the within-subject Factor *Sound Category* and *Picture Category* revealed a significant main effect of *Sound Category, F*_(2, 42)_ = 161.45, *p* < 0.001, η^2^_*p*_ = 0.89, a significant main effect of *Picture Category, F*_(2, 42)_ = 270.07, *p* < 0.001, η^2^_*p*_ = 0.93, as well as a significant interaction of *Sound* and *Picture Category, F*_(4, 84)_ = 26.53, *p* < 0.001, η^2^_*p*_ = 0.56. Overall, audiovisual presentations with unpleasant pictures were rated as more unpleasant than presentations with neutral or pleasant pictures. Presentations with pleasant pictures were rated as most pleasant, for all comparisons *p* < 0.01. Similarly, audiovisual presentations with unpleasant sounds were rated as more unpleasant than presentations with neutral or pleasant sounds and presentations with pleasant sounds were rated more pleasant than presentations with other sounds, for all comparisons *p* < 0.01.

Following the interaction, audiovisual pairs with pleasant pictures were rated as most pleasant if they were accompanied with a pleasant sound and most unpleasant if they were paired with an unpleasant sound, for all comparisons *p* < 0.01.

**Figure 1:**
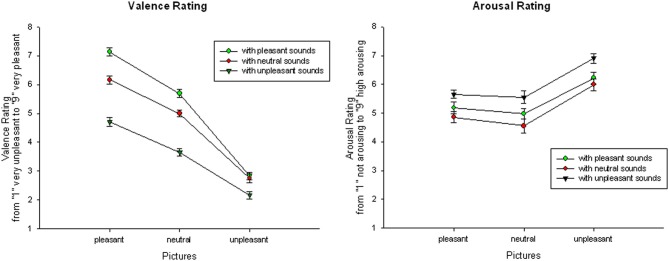
**Valence (left) and arousal (right) ratings for audiovisual presentations: Mean and SEMs of valence and arousal ratings for pleasant, neutral, and unpleasant pictures in combination with pleasant, neutral, and unpleasant sounds**.

Similarly, presentation with neutral pictures were rated as most pleasant if combined with a pleasant and as most unpleasant if they were combined with unpleasant sounds, for all comparisons *p* < 0.01. Presentation with unpleasant pictures were also rated as more unpleasant in combination with an unpleasant sound, for all comparisons *p* < 0.01, but there was no significant difference between unpleasant pictures with neutral or pleasant sounds, *t*_(21)_ = 0.789; *ns*—see Figure 1.

***Congruency***. Comparing the valence ratings of congruent and incongruent audiovisual trials, valence ratings to pleasant pictures with congruent sounds were significantly more pleasant than pleasant pictures with incongruent sounds, *t*_(21)_ = 12.87, *p* < 0.01. Furthermore, valence ratings of unpleasant pictures with congruent sounds were significantly more unpleasant than unpleasant pictures with incongruent sounds, *t*_(21)_ = 7.27, *p* < 0.01.

#### Arousal

***Visual vs. audiovisual condition***. For the arousal ratings we found a significant main effect of *Modality, F*_(1, 21)_ = 18.87, *p* < 0.001, η^2^_*p*_ = 0.47, and a significant main effect of *Stimulus Category, F*_(2, 42)_ = 47.13, *p* < 0.001, η^2^_*p*_ = 0.69, but no significant interaction. Overall, audiovisual presentations were rated as more arousing than pictures only, *t*_(21)_ = 4.34, *p* < 0.01. As expected, unpleasant stimuli were rated as more arousing than neutral stimuli [unpleasant vs. neutral: *t*_(21)_ = 10.36, *p* < 0.01; pleasant vs. neutral: *t*_(21)_ = 2.15, *ns*]. Furthermore, unpleasant stimuli were significant rated as more arousing than pleasant stimuli, *t*_(21)_ = 6.90, *p* < 0.01—see Table 1.

***Audiovisual condition***. For the arousal ratings, a significant main effect of *Picture Category, F*_(2, 42)_ = 43.54, *p* < 0.001, η^2^_*p*_ = 0.68, and a significant main effect of *Sound Category, F*_(2, 42)_ = 37.06, *p* < 0.001, η^2^_*p*_ = 0.64, occurred, but no significant interaction. Overall, stimulus presentations with unpleasant pictures were rated as more arousing than presentations with neutral or pleasant pictures and presentations with pleasant pictures were rated as more arousing than presentations with neutral pictures, for all comparisons *p* < 0.01. Similarly, stimulus presentations with unpleasant sounds were rated as more arousing than presentations with neutral or pleasant sounds, for all comparisons *p* < 0.01, but presentations with pleasant sounds were not rated as significantly more arousing than presentations with neutral sounds, *t*_(21)_ = 1.39, *ns*—see Figure 1.

***Congruency***. Specifically comparing congruent and incongruent stimulus pairs, arousal ratings to pleasant pictures with incongruent sounds were significantly more arousing than with congruent sounds, *t*_(21)_ = 12.46, *p* < 0.01. In contrast, arousal ratings to unpleasant pictures with congruent sounds were significantly more arousing than with incongruent sounds, *t*_(21)_ = 8.39, *p* < 0.01.

### Electrophysiological data

#### P100 component

***Visual vs. audiovisual condition***. For the P100 amplitudes, we found a significant main effect of *Picture Category, F*_(2, 42)_ = 3.70, *p* = 0.041, η^2^_*p*_ = 0.15, and a significant main effect of *Electrode Site, F*_(3, 63)_ = 33.47, *p* < 0.001, η^2^_*p*_ = 0.61, but no other significant main effect or interaction. P100 amplitudes in response to pleasant trials were significant higher than in response to unpleasant trials and there was no significant difference between the visual and audiovisual condition—see Table 2.

**Table 2:** **Mean and standard deviation for the P100 amplitude on parietal (P3,P4) and occipital electrodes (O1,O2) in response to visual and congruent audiovisual presentations**.

**P100**	**Emotion Category**	**Visual *M* (*SD*)**	**Audiovisual *M* (*SD*)**
P3	Pleasant	2.04 (1.67)	3.02 (2.43)
	Neutral	2.16 (2.00)	2.16 (2.71)
	Unpleasant	2.05 (1.91)	2.57 (2.84)
P4	Pleasant	2.86 (1.66)	4.12 (2.38)
	Neutral	2.36 (1.61)	2.90 (2.23)
	Unpleasant	2.89 (1.98)	3.24 (2.28)
O1	Pleasant	6.24 (3.38)	7.29 (4.71)
	Neutral	6.23 (4.31)	6.24 (4.56)
	Unpleasant	6.06 (4.60)	5.77 (4.66)
O2	Pleasant	7.25 (3.40)	8.28 (4.92)
	Neutral	6.76 (4.18)	6.89 (4.49)
	Unpleasant	6.94 (4.51)	6.40 (4.38)

***Audiovisual condition***. For the P100 amplitudes, we found a significant main effect of *Sound Category, F*_(2, 42)_ = 4.803, *p* = 0.014, η^2^_*p*_ = 0.19, and a significant main effect of *Electrode Site, F*_(3, 63)_ = 25.06, *p* < 0.001, η^2^_*p*_ = 0.54, as well as a significant interaction of *Sound Category* and *Electrode Site, F*_(6, 126)_ = 4.04, *p* = .006, η^2^_*p*_ = 0.16. No other main effect or interaction was significant.

**Figure 2:**
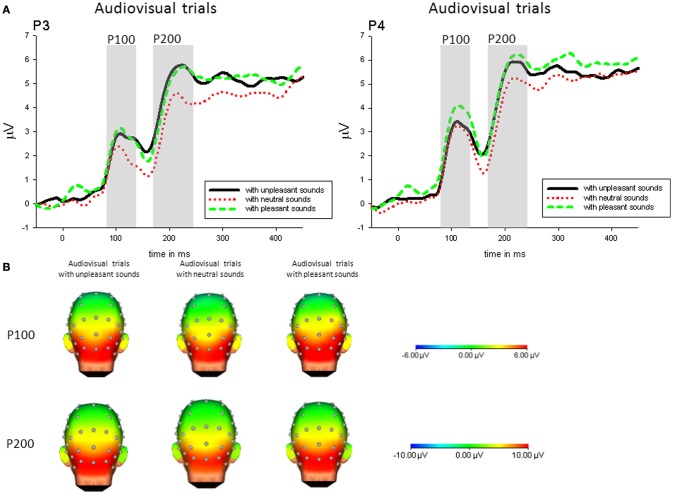
**(A)** ERPs (in μVolt) for representative channels (P3, P4) in response to all pictures (collapsed across unpleasant, neutral, and unpleasant pictures) with pleasant, neutral and unpleasant sounds. Gray boxes show the averaged time interval for the P100 and P200 component. **(B)** Amplitude topography of the P100 (90–120 ms) and P200 (170–230 ms) for audiovisual trials with unpleasant, neural, and pleasant sounds.

Following the interaction, P100 amplitudes on parietal electrodes (P3, P4) were enhanced when pictures were accompanied by pleasant sounds [P3: *F*_(2, 42)_ = 4.86, *p* < 0.05, η^2^_*p*_ = 0.19; P4: *F*_(2, 42)_ = 7.27, *p* < 0.01, η^2^_*p*_ = 0.26] compared to pictures with neutral sounds, whereas this effect was not significant on central electrodes. Additionally, P100 amplitudes to pictures with unpleasant sounds compared to neutral sounds were enhanced on P4 [*t*_(21)_ = 3.23, *p* < 0.01]—see Figure 2.

**Figure 3:**
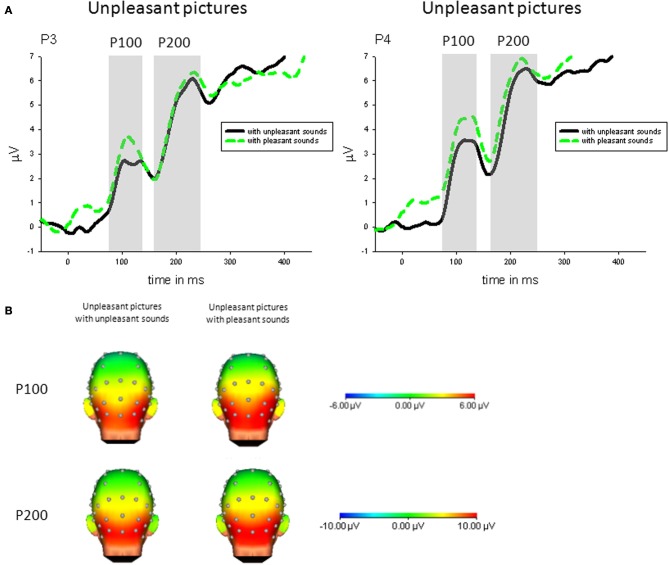
**(A)** ERPs (in μVolt) for representative channels (P3, P4) in response to unpleasant pictures with pleasant and unpleasant sounds. Gray boxes show the averaged time interval for the P100 and P200 component. **(B)** Amplitude topography of the P100 (90–120 ms) and P200 (170–230 ms) for unpleasant pictures with unpleasant and pleasant sounds.

***Congruency***. Specifically comparing congruent and incongruent audiovisual pairs, parietal P100 (P4) was enhanced in response to unpleasant pictures with incongruent (pleasant) compared to unpleasant pictures with congruent sounds, *t*_(21)_ = 2.93, *p* < 0.01—see Figure 3.

#### P200 component

***Visual vs. audiovisual condition***. For the P200 amplitudes, we found a significant main effect of *Modality, F*_(1, 21)_ = 4.44, *p* = 0.047, η^2^_*p*_ = 0.18, a significant main effect of *Stimulus Category, F*_(2, 42)_ = 3.80, *p* = 0.034, η^2^_*p*_ = 0.15, and a significant main effect of *Electrode Site, F*_(3, 63)_ = 69.07, *p* < 0.001, η^2^_*p*_ = 0.77, but no other significant main effect or interaction. P200 amplitudes in response to audiovisual trials were significantly enhanced compared to unimodal picture trials, *t*_(21)_ = 2.11, *p* < 0.05. Furthermore, independent of *Modality*, unpleasant stimulus presentations elicited stronger P200 amplitudes than neutral presentations, *t*_(21)_ = 2.77, *p* < 0.05—see Table 3.

**Table 3:** **Mean and standard deviation for the P200 amplitude on parietal (P3,P4) and occipital electrodes (O1,O2) in response to visual and congruent audiovisual presentations**.

**P200**	**Emotion Category**	**Visual *M* (*SD*)**	**Audiovisual *M* (*SD*)**
P3	Pleasant	3.96 (2.44)	4.52 (1.95)
	Neutral	4.43 (2.84)	3.79 (2.47)
	Unpleasant	4.79 (2.64)	4.81 (2.97)
P4	Pleasant	4.71 (2.65)	5.05 (2.99)
	Neutral	4.54 (2.44)	4.27 (2.77)
	Unpleasant	5.01 (2.90)	5.13 (2.81)
O1	Pleasant	−2.08 (2.30)	−1.49 (2.30)
	Neutral	−1.86 (1.91)	−1.55 (2.02)
	Unpleasant	−1.82 (2.26)	−1.22 (2.00)
O2	Pleasant	−2.26 (2.63)	−1.89 (1.94)
	Neutral	−2.69 (2.73)	−1.12 (2.04)
	Unpleasant	−2.15 (2.41)	−1.51 (1.79)

***Audiovisual condition***. For the P200 amplitudes, we found a significant main effect of *Sound Category, F*_(2, 42)_ = 6.752, *p* = 0.004, η^2^_*p*_ = 0.24, a significant main effect of *Electrode Site, F*_(3, 63)_ = 57.11, *p* < 0.001, η^2^_*p*_ = 0.73, as well as a significant interaction of *Sound Category* and *Electrode Site, F*_(6, 126)_ = 11.31, *p* < 0.001, η^2^_*p*_ = 0.35. No other main effect or interaction was significant.

Following the interaction, P200 amplitudes on parietal electrodes (P3, P4) were enhanced when pictures were accompanied by emotional sounds [P3: *F*_(2, 42)_ = 15.52, *p* < 0.01, η^2^_*p*_ = 0.43; P4: *F*_(2, 42)_ = 12.36, *p* < 0.01, η^2^_*p*_ = 0.37], whereas this effect was not significant on central electrodes—see Figure 2.

***Congruency***. Specifically comparing congruent and incongruent stimulus pairs, parietal P200 (P4) was enhanced in response to unpleasant pictures with incongruent (pleasant) compared to unpleasant pictures with congruent sounds, *t*_(21)_ = 2.32, *p* < 0.05—see Figure 3.

#### Late positive potential (LPP)

***Visual vs. audiovisual condition***. For the LPP, we found a significant main effect of *Stimulus Category, F*_(2, 42)_ = 7.50, *p* = 0.002, η^2^_*p*_ = 0.263. No other main effect or interaction was significant. The LPP in response to unpleasant trials was significantly enhanced compared to neutral, *t*_(21)_ = 2.64, *p* < 0.05, or pleasant presentations, *t*_(21)_ = 2.95, *p* < 0.05—see Table 4.

**Table 4:** **Mean and standard deviation for the late positive potential (LPP) on CP1 and CP2 in response to visual and audiovisual presentations separately for pleasant, neutral and unpleasant presentations**.

**LPP**	**Picture Category**	**Visual *M* (*SD*)**	**Audiovisual *M* (*SD*)**
CP1	Pleasant	2.39 (1.18)	2.57 (1.30)
	Neutral	1.96 (1.05)	1.99 (0.96)
	Unpleasant	2.78 (1.83)	3.45 (2.15)
CP2	Pleasant	2.36 (1.34)	2.67 (1.33)
	Neutral	1.86 (0.98)	1.90 (0.91)
	Unpleasant	3.00 (1.73)	3.65 (1.99)

**Figure 4:**
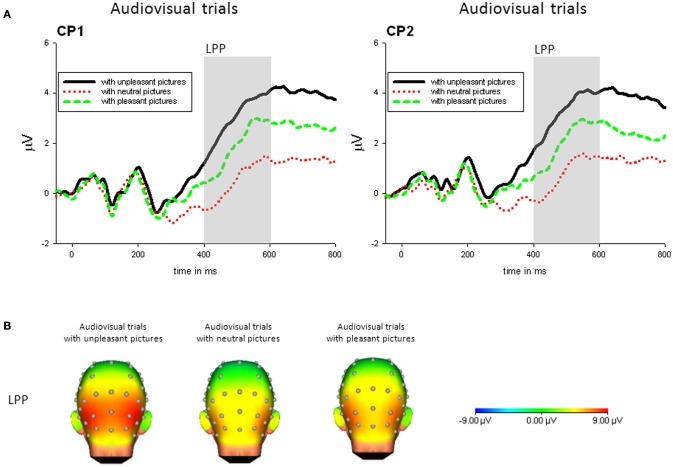
**(A)** ERPs (in μVolt) for representative channels (CP1, CP1) in response to audiovisual trials with pleasant, neutral and unpleasant pictures (collapsed across unpleasant, neutral, and unpleasant sounds). Gray boxes show the averaged time interval for the LPP component. **(B)** Amplitude topography of the LPP (400–600 ms) for audiovisual trials with unpleasant, neural, and pleasant pictures.

***Audiovisual condition***. For audiovisual trials, there was a significant main effect of *Picture Category, F*_(2, 42)_ = 13.95, *p* < 0.001, η^2^_*p*_ = 0.399. No other main effect or interaction was significant. The LPP in response to trials with unpleasant pictures was significantly enhanced compared to trials with neutral, *t*_(21)_ = 3.99, *p* < 0.01, or pleasant pictures, *t*_(21)_ = 3.70, *p* < 0.01. Furthermore, in response to presentations containing pleasant pictures compared to neutral pictures an enhanced LPP was found, *t*_(21)_ = 2.91, *p* < 0.05—see Figure 4.

***Congruency***. For the LPP, there was no significant difference between congruent and incongruent trials, all *p*s > 0.19.

## Discussion

The present study investigated the impact of concurrent emotional sounds on picture processing. Extending previous research on emotional face-voice pairings, the utilized stimulus material (pictures and sounds) covered a wide range of semantic contents (Bradley and Lang, 2000; Lang et al., 2008). Results showed that high arousing unpleasant compared to neutral pictures were associated with an increased parietal P200 and a more pronounced centro-parietal LPP regardless of the accompanying sound. For audiovisual stimulation, increased parietal P100 and P200 amplitudes were found in response to all pictures which were accompanied by unpleasant or pleasant sounds compared to pictures with neutral sounds. Most importantly, parietal P100 and P200 were enhanced in response to unpleasant pictures with incongruent (pleasant) compared to congruent sounds. Additionally, subjective ratings clearly showed that both emotional information—sounds and pictures—revealed a significant impact on valence and arousal ratings.

Regarding the neural processing, indicators of selective processing of emotional compared to neutral pictures were replicated. Independent of the accompanying sound, unpleasant compared to neutral pictures were associated with an increased P200 and a more pronounced LPP. These findings are in line with studies reporting that unpleasant stimuli were associated with an enhanced P200 which is thought to originate in the visual association cortex and reflect enhanced attention toward unpleasant picture cues (Carretie et al., 2001a,b, 2004). Similarly, the LPP was more pronounced in response to unpleasant pictures compared to neutral indicating sustained processing and enhanced perception of high arousing material (Schupp et al., 2000; Brown et al., 2012). Most recent research reported enhanced LPP amplitudes to both, high arousing pleasant and unpleasant stimuli (Cuthbert et al., 2000; Schupp et al., 2000). In the current study, the lack of enhanced LPP amplitudes for pleasant pictures might be explained in terms of emotional intensity. Thus, pleasant pictures (and audiovisual pairs containing pleasant pictures) were rated as less arousing than unpleasant pictures (and audiovisual pairs containing unpleasant pictures).

Comparing visual and audiovisual stimulation, pictures with preceding congruent sounds were associated with enhanced P200 amplitudes regardless of picture and sound valence compared to pictures without sounds. This may be interpreted as an enhanced attentional allocation to the pictures when they were accompanied by congruent sounds. Similarly, rating data revealed that audiovisual pairs were perceived as more arousing and more emotional intense than visual stimuli alone. Thus, the enhanced P200 might reflect an increased salience of a picture when it is accompanied by a (congruent) sound. Consequently, pictures with sounds seem to receive a higher salience in contrast to pictures without sounds. Generally, the finding of altered P200 amplitude is in line with previous studies on multimodal information (see also Jessen and Kotz, 2011). However, in contrast to the present finding of enhanced P200 for multimodal information, several studies reported reduced P200 amplitudes to multimodal compared to unimodal stimulation in multimodal human communication (Stekelenburg and Vroomen, 2007; Paulmann et al., 2009). This has been interpreted as an indicator of facilitated processing of multimodal redundant information and state that multimodal emotion processing is less effortful than unimodal processing. However, variant findings may relate to methodological differences regarding the stimulus material (faces and voices vs. more complex stimuli), focus of analyses (auditory or visual evoked potentials) and order and timing of the presentation (simultaneous vs. shifted presentation of sound and pictures). As (congruent) sound and picture stimuli did not transport redundant but additional information in the current study (cf. face-voice pairings), the present findings of generally enhanced responses to multimodal stimuli may rather reflect intensified salience detection than a facilitated processing.

Regarding the specific findings for audiovisual stimulation, an increased parietal P100 and an increased P200 was observed in response to all pictures which were accompanied by unpleasant or pleasant sounds compared to pictures with neutral sounds. The modulation of early visual components as the P100 by emotional sounds may be interpreted as evidence that emotional sounds may unspecifically increase sensory sensitivity or selective attention to consequently improve perceptual processing of all incoming visual stimuli (Mangun, 1995; Hillyard et al., 1998; Kolassa et al., 2006; Brosch et al., 2009). Likewise, the increased P200 amplitude to all pictures which came along with emotional sounds could be interpreted as an unspecific enhancement of attentional resources toward the visual stimuli if any emotional information was conveyed by the sounds. Both P100 and P200 may reflect an important mechanism to support fast discrimination between relevant and irrelevant information (in all sensory channels) and thus to prepare all senses for following relevant information in order to facilitate rapid and accurate behavioral responses (Öhman et al., 2001, 2000).

Of particular interest, the emotional mismatch of visual and auditory stimuli revealed a pronounced impact on picture processing. Specifically, a reduction of P100 and P200 amplitudes was observed for unpleasant pictures with congruent (unpleasant) compared to incongruent (pleasant) sounds. This finding indicates that unpleasant pictures processing is facilitated when they were preceded by congruent unpleasant sounds. In contrast, the incongruent combination (unpleasant picture and pleasant sounds), may require more attentional resources as indicated by enhanced P100 and P200 responses. This finding is in line with previous research on emotional perceptual integration suggesting facilitated processing for emotional congruent information (de Gelder et al., 1999; Pourtois et al., 2002; Meeren et al., 2005). Regarding the question why an incongruency effect was only found for unpleasant pictures paired with pleasant sounds, we can only speculate that this mismatch is much more behaviorally relevant as the opposite one (pleasant picture with unpleasant sound). The sudden onset of an aversive visual event after pleasant sounds might indicate that immediate change of behavior is needed to avoid potential surprising harm. However, when there is an aversive sound present but then the visual signal provides information which is non-threatening, this is not as arousing and relevant for the organism to change behavior at the onset of the visual event. All the more, this finding also warrants further research on the timing and order of multi-modal affective stimulation.

Subsequent processing stages of the pictures were not modulated by concurrent emotional sounds. Specifically, LPPs to unpleasant picture did not vary as a function of picture-sound congruency in the present study. These findings contrast with a recent study reporting later visual processing modulated by congruent auditory information (Spreckelmeyer et al., 2006). However, future studies will need to integrate crossmodal resource competition (cf. Schupp et al., 2007, 2008).

Regarding the underlying brain structures, our results are in line with functional imaging data suggesting that multisensory interaction takes place in posterior superior temporal cortices (Pourtois et al., 2005; Ethofer et al., 2006a). Furthermore, recent fMRI studies suggested that emotional incongruence is accompanied with higher BOLD-responses (e.g., in a cingulate-fronto-parietal network) compared to congruent information (Müller et al., 2011, 2012b). However, further studies reported enhanced neural activation in response to congruent compared to incongruent information (Spreckelmeyer et al., 2006; Klasen et al., 2011; Liu et al., 2012). Thus, future studies are needed to clarify whether congruent information is processed in a facilitated or intensified fashion and which brain regions are significantly involved in these processes.

Complementary findings are provided by verbal report data. Similar to the ERP findings, a congruency effect specifically pronounced for unpleasant picture materials with unpleasant sounds was revealed for arousal ratings. Specifically, more pronounced arousal was reported for unpleasant pictures with congruent as compared to incongruent sounds. Further, pleasant picture ratings were generally lower in arousal. In addition, valence congruence revealed lower arousal ratings in comparison to pleasant pictures with unpleasant sounds. Accounting for that difference between unpleasant and pleasant pictures, an evolutionary perspective may be of particular relevance. From a survival point of view, the detection of possibly threatening visual information is much more relevant (Öhman and Wiens, 2003) when the auditory domain prompts the anticipation of unpleasant stimulation. Conversely, the violation of anticipated pleasant visual information triggered by unpleasant sounds appears behaviorally less momentous.

## Limitations

Several limitations of the present study need to be acknowledged. Regarding congruency effects, the present study focused on emotional rather than on semantic mis/match. Accordingly, picture and sound stimuli were not specifically balanced with regards to their semantic content. For example, pictures depicting animals could be accompanied by human or environmental sounds and vice versa. Consequently, a systematic differentiation between emotional and sematic (in)-congruency cannot be inspected in the present study. Further, as for other studies, the question occurs whether the present findings actually reflect multimodal integration of emotional information (Ethofer et al., 2006b) or rather enhancement effects due to increased (emotional) intensity of audiovisual compared to unimodal stimuli. To elucidate this question in detail, future studies will need to systematically vary emotional intensity during unimodal and multimodal presentations.

Furthermore, it is important to mention that our comparison of visual and audiovisual stimuli is to be seen with caution. In line and to be comparable with several existing studies on multimodal emotion processing (e.g., Pourtois et al., 2000, 2002; Müller et al., 2012a), we defined the baseline to 100 ms preceding the multimodal stimulation (picture onset) which is favorable because (1) it is as close as possible to the relevant time epoch and therefore corrects for relevant potential level shifts and (2) it subtracts audio-evoked brain activity and therefore multimodal effects are less confounded. However, for comparison of multimodal vs. visual only, this baseline definition corrects for a pure double-stimulation effect in the multimodal condition but the different stimulation during the baseline might lead to incommensurable effects. Future studies could investigate this with adequate experimental paradigms.

## Conclusion

The present study support the notion of multimodal impact of emotional sounds on affective picture processing. Early components of visual processing (P100, P200) were modulated by the concurrent presentation of emotional sounds. Further, the congruence of sound and picture materials was important, especially for unpleasant picture processing. In contrast, later indices of facilitated processing of emotional pictures (LPPs) remained relatively unaffected by the sound stimuli. Taken together, further evidence is provided for early interactions of multimodal emotional information beyond human communication.

### Conflict of interest statement

The authors declare that the research was conducted in the absence of any commercial or financial relationships that could be construed as a potential conflict of interest.

## Footnotes

^1^From originally 27 participants, *N* = 5 were excluded due to technical problems or extensive artifacts.

^2^Between male and female participants there were no differences except for age: male participants, *M* = 22.55, *SD* = 3.50, were slightly older than female participants, *M* = 20.09, *SD* = 1.22, *t*_(21) = 2.19; *p* = 0.04.

^3^Nos. of the selected pictures from the IAPS: *pleasant*: 2071, 2165, 2224, 2344, 2501, 4250, 4599, 4607, 4659, 4681, 8030, 8461, 8540, 1812, 5831, 5551, 5910, 7280, 8170, 8502; *unpleasant*: 3000, 3005.1, 3010, 3053, 3080, 3150, 3170, 3350, 6230, 6350, 6360, 6510, 9250, 9252, 9902, 9921, 6415, 9570, 6300; *neutral*: 2372, 2385, 2512, 2514, 2516, 2595, 2635, 2830, 7493, 7640, 1675, 5395, 5920, 7037, 7043, 7170, 7207, 7211, 7242, 7487. Nos. of selected sounds from the IADS: *pleasant*: 110, 112, 200, 202, 220, 226, 230, 351, 815, 816, 150, 151, 172, 717, 726, 809, 810, 813, 817, 820; *unpleasant*: 241, 242, 255, 260, 276, 277, 278, 284, 285, 286, 290, 292, 296, 105, 422, 501, 600, 703, 711, 713; *neutral*: 246, 262, 361, 368, 705, 720, 722, 723, 113, 152, 171, 322, 358, 373, 374, 376, 382, 698, 700, 701.

^4^The edited sounds were preliminary tested for valence and arousal in a separate pilot study and this unpublished pretest showed that a presentation duration of 2 s is adequate to elicit emotional reactions comparable to the original sounds.

^5^Originally, the experimental part also comprised 60 unimodal trials with unpleasant, neutral and pleasant sounds. As the analysis focused on visual ERPs only, these trials were not considered for further analysis.

^6^No processing differences were observed at PO9/10 within the EPN time window.

^7^For the P100, four individual electrodes were entered into the ANOVA (P3, O1, P4, O2), for the P200 the electrodes P3, C3, P4, and C4 and for the LPP, CP1, and CP2 were entered.
